# Bond Properties of Carbon Fiber Reinforced Polymer and Corrosion-Cracked Reinforced Concrete Interface: Experimental Test and Nonlinear Degenerate Interface Law

**DOI:** 10.3390/ma14185333

**Published:** 2021-09-15

**Authors:** Yongzhi Gong, Yingjie Shan, Yuyuan Wu, Liping Wang, Xiaojie Liu, Faxing Ding

**Affiliations:** 1 College of Civil Engineering, Central South University, Changsha 410075, China; gyzcsu@csu.edu.cn (Y.G.); wlp2016@csu.edu.cn (L.W.); liuxj@csu.edu.cn (X.L.); dinfaxin@csu.edu.cn (F.D.); 2 Huidong County Construction Quality Inspection Station, Huizhou 516000, China; yy.wu@hotmail.com

**Keywords:** corrosion, CFRP to concrete interface, double shear lap test, effective bond length, nonlinear degenerate law

## Abstract

Existing experimental research on bond properties of the interface between Carbon Fiber Reinforced Polymer (CFRP) and damaged concrete is limited, although CFRP strengthening technology has been widely used for corroded reinforced concrete structures. This work investigated the bond behavior of CFRP to the corrosion-cracked concrete interface, in which three factors were considered for experimentation, including corrosion degree, concrete strength and concrete cover thickness. The tests were conducted by developing a self-balancing double shear lap test device. In addition, a corrosion scene was provided simultaneously to simulate the external corrosion environment. The results showed that three peeling modes of CFRP sheets were observed with respect to corrosion degrees of the steel bars. The effects of the three factors on the stripping bearing capacity and effective bond length of CFRP sheets were discussed by systematic parametric analysis. Finally, a nonlinear degenerate law of CFRP-to-concrete interface considering the corrosion degree was improved and verified in this study.

## 1. Introduction

In coastal areas or inland lakes with high salinity, the steel bars in reinforced concrete (RC) structures are easy to rust by chloride and carbon dioxide due to the porosity of concrete, which will lead to a reduction in load-carrying capacity and durability of RC components, as well as huge economic loss. For sustainable development, externally bonded Carbon Fiber Reinforced Polymer (CFRP) sheets have received increasing attention in retrofitting corroded RC structures [1]. In this technology, CFRP sheets are only applied to the concrete surface after the external surface of the concrete is prepared by grinding, sandblasting or high-pressure water spraying. One of the main drawbacks of this technology is that CFRP attachments may debond from the adjacent concrete surface. Therefore, the CFRP-to-concrete interface is the critical but vulnerable area for stress transmission, which affects the effectiveness of this technique.

Many studies about the mechanical properties of the interface between Fiber Reinforced Polymers (FRP) and concrete have been carried out in recent years [2,3,4,5,6,7,8,9,10,11,12,13,14,15], and the parameters that significantly affect the bond behavior have been identified, including concrete grade, concrete cover thickness, properties of the epoxy binder, exposure conditions and so on. Due to the excellent corrosion resistance and environmental durability of CFRP materials, externally bonding CFRP sheets are widely used to repair offshore RC elements. Furthermore, many researchers have been investigating the impact of the simulated marine environment on bond properties of CFRP-to-concrete interface [16,17,18,19,20]. They investigated the deterioration effects of moisture conditions and the dry-wet cyclic conditions of salt solution on the durability of the bond behavior between CFRP and concrete interface. However, a more critical factor is the corrosion of steel bars. Severe corrosion can induce the cracking of concrete, thus undermining the bond behavior of FRP to concrete interface. However, the study of bond behavior between CFRP sheets and corrosion-cracked concrete surface is limited. Haddad et al. [20,21,22] conducted experiments on this gap and found that corrosion-induced cracking caused a significant reduction in bond strength, slippage at ultimate stress and bond stiffness. However, the design scheme of the specimens in their study did not accurately simulate the actual CFRP strengthening scheme. Moreover, there is no degradation model considering corrosion for the effective bond length or interface law between CFRP sheets and corrosion-cracked concrete surface. Hence, a standardized test method is required to investigate the bond behavior between corrosion-cracked concrete and CFRP sheets in the marine environment with consideration of other important parameters, such as concrete grade and concrete cover thickness.

In this study, electro-chemical accelerated corrosion test and double shear lap test were carried out to study the combined effect of corrosion degree, concrete strength and concrete cover thickness on bond behavior of CFRP to corrosion-cracked concrete interface. Based on existing theoretical models, the factor of corrosion degree was introduced to obtain the degradation model of effective bond length and bond stress-slip relationship. The understanding gained will help in interpreting durability-oriented CFRP applications in the marine environment.

## 2. Experimental Program

### 2.1. Specimens Preparation

Table 1 shows an outline of the test specimen. There were a total of 17 specimens in this test, which can be subdivided into three series: Series C30-20, Series C30-40 and Series C50-40. Specimen label is named as “CM-X-Y”, where M means concrete grade set as 30 and 50. X means the concrete cover thicknesses set as 20 mm and 40 mm. Y refers to the target corrosion degree with values of 3%, 5%, 7%, 10% and 15%, respectively. The bond behavior between CFRP and intact concrete is less affected by the concrete cover thickness; thus, only one specimen (C30-20-0) was set in C30 series.

**Figure 1:**
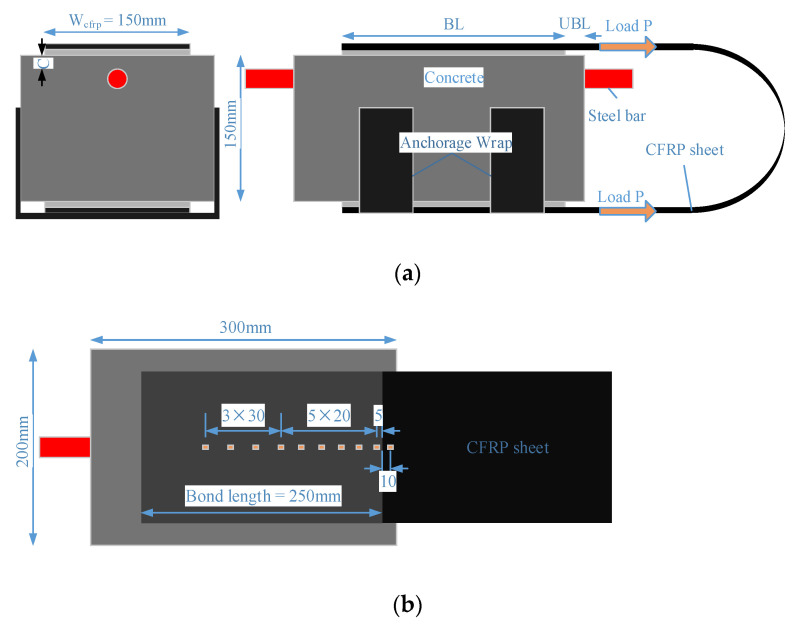
Double shear lap test specimens with detailed dimensions: (**a**) side view; (**b**) top view.

The specimen configuration is shown in Figure 1a. A steel bar with the length of 400 mm was embedded near the top surface of the concrete prism (150 mm × 200 mm × 300 mm), with both ends extending out of specimens by 50 mm. A single layer of 150 mm-wide CFRP sheet was bonded on two opposite sides of the concrete prism along the axial direction. An anchorage wrap was attached to the bottom CFRP sheets to ensure the full development of bond performance between CFRP and corrosion-damaged concrete. The bond length (BL) was 250 mm, which is longer than the effective bond length (EBL) estimated from the model proposed by Chen and Teng [23]. An unbonded length (UBL) of 20 mm was reserved to avoid an unfavorable boundary effect, which has been verified by Mazzotti et al. [24] with FE numerical study.

### 2.2. Material Properties

The selected raw materials for C30 concrete were ordinary Portland cement whose level is 42.5 grade, crushed stone (particle size in the range of 5–25 mm), medium sand and tap water. The mix proportions of C30 were cement:sand:aggregate = 1:1.49:3.17 with a water-to-cement ratio of 0.5, and the mix proportions of C50 were cement:sand:aggregate = 1:1.5:2.92 with a water-to-cement ratio of 0.41. These two mixtures of concrete had a mean compressive strength of 40.88 MPa and 53.21 MPa, respectively, determined on standard concrete cubes (150 mm × 150 mm × 150 mm), after a 28-day standard curing condition.

HRB335 crescent rib steel bar with a diameter of 18 mm was used in this test. This bar has the yield strength of 335 MPa, the tensile strength of 455 MPa and Young’s modulus of 200 GPa.

CFRP sheets used in this study were produced by Shanghai tongzheng building materials Company. The adhesive was a mixture of glue A and B produced by Yueqing Zhonggu Construction Technology Development Company with a ratio of 1:0.4. The mechanical properties of these two items provided by the manufacturers are shown in Table 2.

### 2.3. Manual Operation Details for Bonding CFRP Sheets

Fifteen corrosion specimens and two control specimens were bonded with CFRP sheets according to the manufacturer’s instructions. To ensure the bonding quality of CFRP sheets, the loose concrete layer in the bonded area was removed with a sander. After washing and wiping, a layer of adhesive was evenly coated on this prepared area with a plastic scraper, and then a CFRP sheet was put on it. The second layer adhesive was evenly brushed on the surface of CFRP sheet to completely soak it. The indoor temperature was 20–25 °C, and the curing time was set as 7 days to render full development of the adhesive strength. In the process of glue curing, the surface of CFRP sheets was pressed by hand. If there is a large hollowing area, re-apply glue and re-stick the CFRP sheets.

### 2.4. Electro-Chemical Accelerated Corrosion Simulation

#### 2.4.1. Determination of Corrosion Rate

Based on Faraday’s Law, the target corrosion degree ($\rho_{s}$) was controlled by corrosion time (*t*) [25]:

(1) $$t=13441\cdot D\cdot (1-\sqrt{1-\rho_{s}})/i$$

where i is current density, which was set as 0.02 A/cm^2^; D is diameter of steel bars in cm; t is the corrosion time in second. The required corrosion time of five target corrosion levels are 18,284 s, 30,630 s, 43,107 s, 62,077 s and 94,411 s, respectively.

The actual corrosion degree of reinforcement (η) is expressed by Equation (2).

(2) $$\eta =m_{0}-m/m_{0}\times 100\%$$

where $m_{0}$ is the initial weight of steel bars; m is the remaining weight of it after removing corrosion medium.

#### 2.4.2. Details of Implementation of Accelerated Corrosion Method

Specimens with target corrosion degree were soaked in NaCl solution with a concentration of 5% for 5 days, then connected in parallel with the circuit as anodes with a copper sheet as cathode. They were corroded to 0.5% before bonding CFRP sheets, then put back into the tank to corrode to the target degree. The purpose of this operation is to simulate that RC members with slight corrosion cracks would be put back into service again in the marine environment after being strengthened with CFRP sheets. Non-corrosive specimens (C30-20-0 and C50-40-0) were put in the same temperature environment but not soaked in salt water.

### 2.5. Layout of Strain Gauges

A total of 10 strain gauges were axially arranged along the centerline of CFRP sheets: one was placed at the unbounded zone, and nine were placed at the bonded zone (Figure 1b).

### 2.6. Double Shear Lap Test

**Figure 2:**
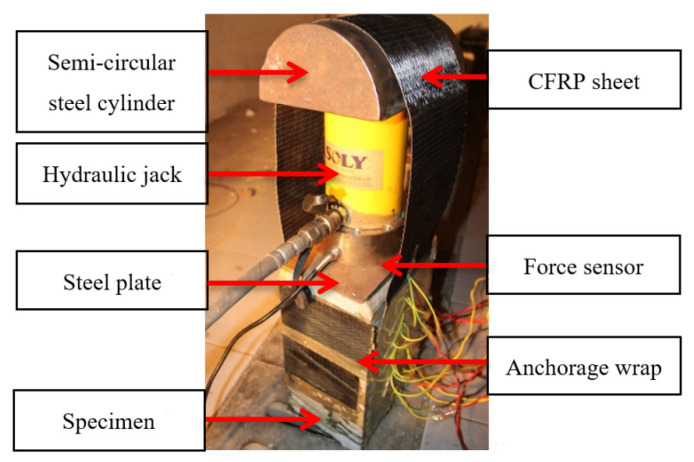
The self-balancing double shear lap test loading device.

Mukhtar et al. [26] presented a review of five methods of testing bond behavior of FRP-concrete interface, including single shear lap test, double shear lap test, bending type test, mixed-mode type test and direct tension pull-out test. They thoroughly analyzed the advantages and disadvantages of each experimental device, and proposed a new double shear lap testing configuration, which inspired authors to design a self-balancing double shear device to study the shear bond performance between FRP and concrete, as shown in Figure 2. Different from the complicated stress state of FRP-concrete interface in bending type test and mixed-mode type test, only shear stress exists in FRP-concrete interface in double shear lap test. Besides, the proposed self-balancing double shear device can avoid the problem of the insufficient development of strength of CFRP sheets due to premature debonding in the common single shear lap test.

As shown in Figure 2, a hydraulic jack and a force sensor were placed between the specimen and a semi-round steel cylinder. The CFRP sheet was twined on this cylinder, which can increase the contact area between the hydraulic jack and CFRP sheet. To avoid stress concentration, a steel plate was placed between the force sensor and the concrete specimen. This feasible device can avoid the problem of uneven stress on both sides of CFRP sheets, which is common in conventional double shear tests.

Pre-loading method was adopted to ensure that two surfaces of CFRP sheet were parallel and the force acted on the center of specimens. Static loading method controlled by force was adopted with 1 kN loading grade and 3 min holding time. The test was terminated when CFRP sheets were peeled off or the self-balancing loading device could not sustain balance due to large-area peeling of the CFRP sheets.

## 3. Test Result and Discussion

### 3.1. Results of Electro-Chemical Accelerated Corrosion Test

**Figure 3:**
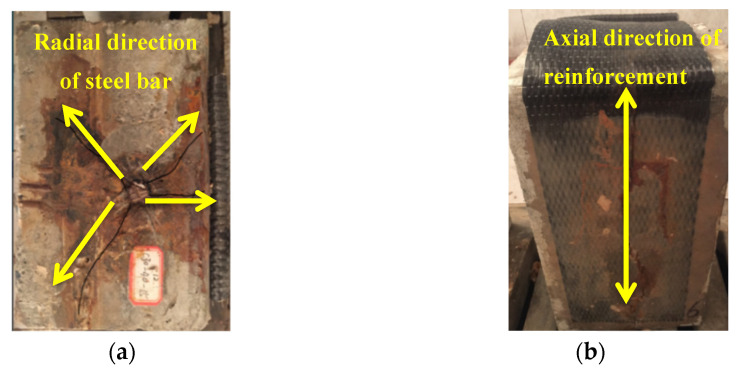
Observations from the accelerated corrosion test: (**a**) Radial propagated crack(C30-40-15); (**b**) Rust-dyed CFRP sheets(C30-40-15).

Cracks propagated along the radial direction of reinforcement (Figure 3a). From the top view, a red trace along the axial direction of the steel bar can be seen on CFRP sheets (Figure 3b). Due to the combined negative effect of salt water immersion [27] and internal transverse tensile stress caused by concrete cracking, the adhesive layer cracked and the red corrosion medium soaked in chlorinated water, penetrated through the CFRP sheet and dyed it.

After the double shear lap test, the corroded steel bars were taken out from the failed specimens to calculate the actual corrosion degree, as reported in Table 3.

### 3.2. Result Analysis of Double Shear Lap Test

#### 3.2.1. Failure Modes

**Figure 4:**
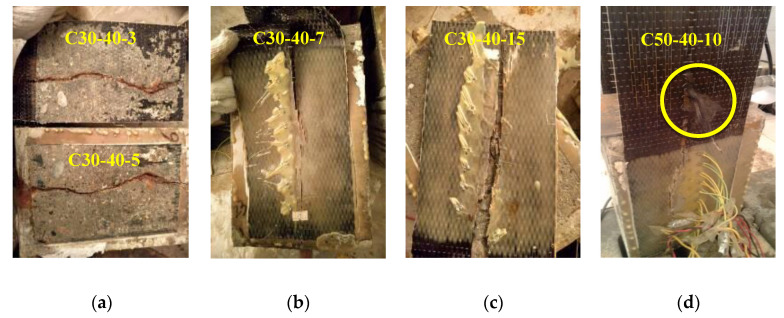
Typical failure modes of specimens: (**a**) Failure mode A; (**b**) Failure mode B; (**c**) Failure mode C; (**d**) Partially teared of CFRP fiber.

The crack initiation load ($P_{cr}$) when CFRP sheet starts to be stripped from the loaded end; the ultimate peeling load ($P_{u}$) when CFRP sheet is peeled off or the device cannot sustain balance; and three peeling modes of CFRP sheets are summarized in Table 3. Mode A (Figure 4a) means the completely peeling off of CFRP sheets with a sound “bang”. Mode B (Figure 4b) means the large-area peeling of CFRP sheets due to stress concentration caused by corrosion-induced cracking of concrete. Tests with this mode were terminated early due to the unbalance of device. Mode C (Figure 4c) indicates that CFRP sheets are torn along the concrete crack and peeled off in pieces. CFRP sheets of C30-20-3 and C50-40-7 were damaged after the corrosion test, so they were not subjected to double shear test.

All non-corrosive specimens and those with corrosion degrees lower than 5% showed failure mode A. Besides, CFRP sheets of two specimens with higher corrosion degrees (8.6% and 11.7%) in the C30-20 series were also completely peeled off. Three specimens with intermediate corrosion degree (6.0%, 6.7% and 9.4%) were failed in mode B, in which bond performance may not be fully developed due to the early termination of the loading process. This indicates that concrete cracking caused by steel corrosion has a negative effect on the bond property of CFRP to concrete interface. For the severely corroded specimens, due to the excessive crack width of concrete and the adverse effect of corrosion medium penetrating into the bond surface, the peeling mode of CFRP sheets was C. Besides, CFRP fibers were partially torn in specimens C30-20-10 and C50-40-10, as shown in Figure 4b, which was caused by the negative effect of internal stresses introduced by moisture absorption of CFRP on the fiber-matrix interface bonding 17.

#### 3.2.2. Effects of Corrosion, Concrete cover Thickness and Concrete Strength on Peeling Strength

**Figure 5:**
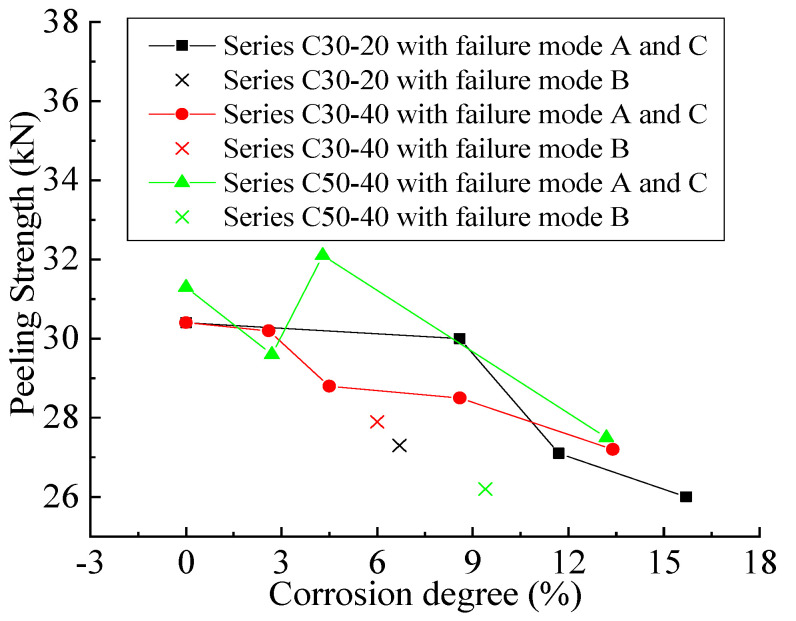
Combined effect of three factors on peeling strength.

The combined effect of three factors on the peeling strength ($P_{u}$) is shown in Figure 5. Data of failure mode B were marked with a cross and discarded because $P_{u}$ may not be fully developed due to the early termination of the test. In Series C30-20 and Series C30-40-3, $P_{u}$ decreases with the increase in corrosion degree, which reflects the negative impact of corrosion-induced cracks on the peeling strength of FRP-concrete interface. In Series C50-40, the peeling strength of specimen C50-40-3 is lower than that of C50-40-0 and C50-40-5, which can be explained by the great variability related to the corrosion phenomenon. In addition, from the comparison results of the three series of specimens, it can be concluded that the concrete cover thickness has no obvious effect on the peeling strength of CFRP sheets for specimens with or without corrosion. For uncorroded specimens, concrete strength has a positive influence on peeling strength.

#### 3.2.3. Strain Profile along the Bond Length and Effective Bond Length (EBL)

**Figure 6:**
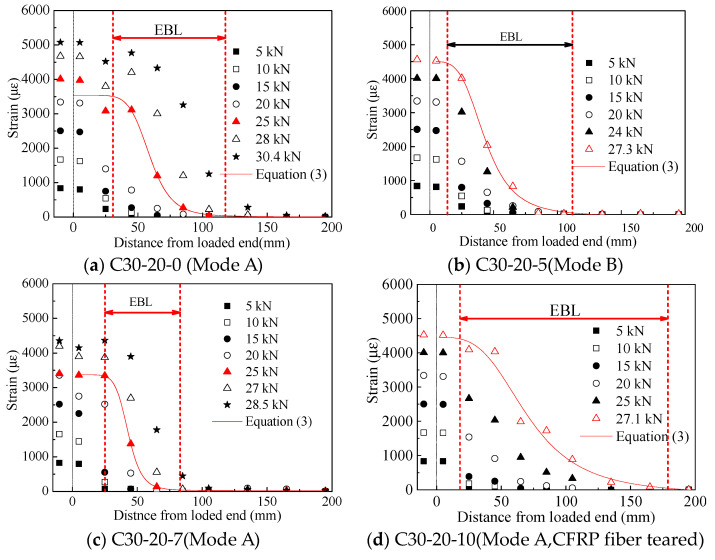

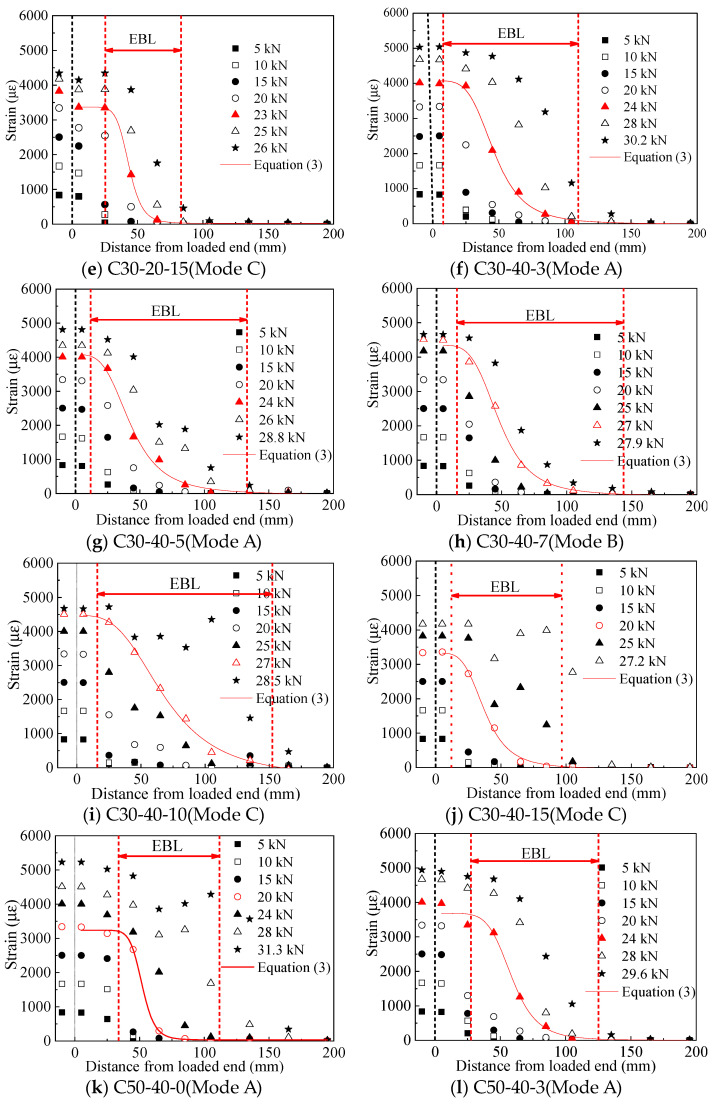

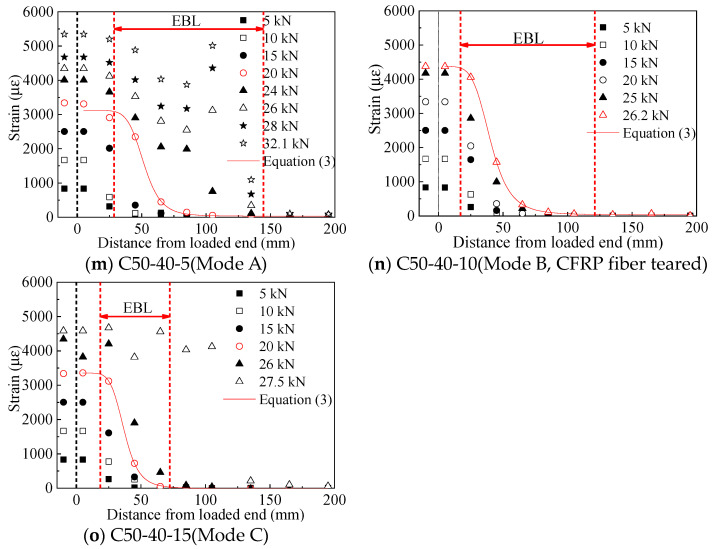
Strain distribution along the bond length of specimens (**a**) C30-20-0 with failure mode A; (**b**) C30-20-5 with failure mode B); (**c**) C30-20-7 with failure mode A; (**d**) C30-20-10 with failure mode A; (**e**) C30-20-15 with failure mode C; (**f**) C30-40-3 with failure mode A; (**g**) C30-40-5 with failure mode A; (**h**) C30-40-7 with failure Mode B; (**i**) C30-40-10with failure mode C; (**j**) C30-40-15 with failure mode C; (**k**) C50-40-0 with failure mode A; (**l**) C50-40-3 with failure mode A; (**m**) C50-40-5 with failure mode A; (**n**) C50-40-10 with failure mode B; (**o**) C50-40-15 with failure mode C.

Strain distribution along the shear direction obtained from strain gauges is shown in Figure 6a–o. The abscissa refers to the distance from the measuring point to the loaded end, and the ordinate represents the corresponding strain values of CFRP. The strain profile shape of each specimen is similar, and the distribution of strain of CFRP sheets along the bond length conforms to the expected law, which proves the reliability and applicability of the designed self-balancing device in this study. To eliminate the effect of the local material variation and stress concentration on the fluctuations in the measured strain, a model is required to fit the distribution of strain along the bond length. Referring to the peeling phenomenon of CFRP sheets and the definition of EBL, this model should meet the following requirements: (1) the tangent line of the curve is 0 at the loaded end, which means that the CFRP sheet is initially peeled off at the loaded end; and (2) the tangent line of the curve is 0 at the free end, which means that the CFRP sheet is stuck well at the free end, thus obtaining a reliable EBL. Fitting result using software Origin 8.0 shows that Equation (3) can not only meet the above requirements, but is also better fit with the strain profile than the Unary Cubic Equation proposed in [28].

(3) $$\varepsilon (x)=\left(a-b\right)/\left[1+{\left(x/x_{0}\right)}^{c}\right]+b$$

where a, b, c and $x_{0}$ are the coefficients obtained by fitting, and x is the distance to the loaded end. EBL obtained from these profiles is summarized in Table 3, which was defined as the distance between the points corresponding to 99% and 1% of the strain at the loaded end when the strain profile at the crack face tends to be plateau [28].

Figure 6 shows that the value of the first strain gauge near the loaded end in the bonded area is almost the same as that in the unbonded area. For specimens with failure modes A and C, strain profile shape changes from concave to convex with the load increasing, and a platform is formed at the loaded end, which indicates the initial peeling position of CFRP sheets. For specimens with failure mode B, the shape of the strain curve does not fully develop into convex, and no plateau is formed at the loaded end. Therefore, EBL obtained from this mode is smaller than the actual EBL value. For specimens with failure modes A and C, the load level for determining EBL is about 60–90% of the maximum load, while for specimens with failure mode B, the load level for determining EBL is about 100% of the maximum load. This result shows the more brittle character of failure mode B than the other two failure modes.

## 4. Parametric Analysis on EBL and Bond Stress-Slip Relationships Considering Corrosion

### 4.1. EBL Model Considering Corrosion

F. Chen et al. [23] proposed a model to obtain EBL ($L_{e}$), as shown in Equation (4), which has been cited by the American Guideline [29], the accuracy of which has been proved by many scholars [30,31].

(4) $$L_{e}=\sqrt{E_{f}\cdot t_{f}/\sqrt{f_{c}^{’}}}$$

where $f_{c}^{’}$ is the compressive strength of a cylinder standard specimen with a diameter of 6 inches and a height of 12 inches. $t_{f}$ and $E_{f}$ are the thickness and elastic modulus of CFRP sheets.

**Figure 7:**
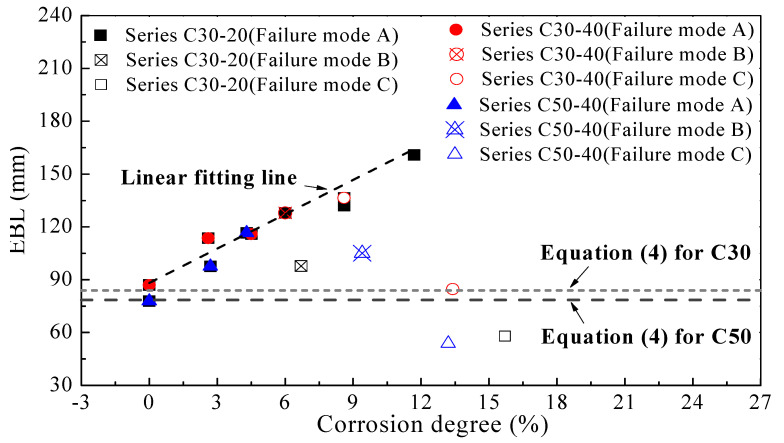
EBL—corrosion degree relationship.

Figure 7 shows the combined effect of concrete grade and corrosion degree, as well as concrete cover thickness on EBL. For benchmark specimens (C30-20-0 and C50-40-0), EBLs obtained from the test results are consistent with those calculated by Equation (4). But for corroded specimens, except those with failure mode C, EBLs obtained from this test are larger than the calculated values, which is due to the lack of consideration of corrosion in Equation (4). As shown in Figure 7, concrete strength and concrete cover thickness have no effect on EBL, but for specimens with corrosion degree of less than 12%, EBL and corrosion rate are linearly related. It can be noted that specimens with failure mode B deviate from this straight line because the bond performance has not been fully developed. For specimens with corrosion degree of larger than 12%, for which failure mode is C, EBLs are smaller than that of benchmark specimens. This is because of the character of mode C, which can be considered that two pieces of CFRP sheets are respectively adhered to the concrete surface without crack, and due to the adverse effect of immersion in saline water and dispersion of corrosion medium on the CFRP to concrete interface. So EBLs of specimens with failure mode C are smaller than that of benchmark specimens.

Data of specimens with failure mode C were removed for proposing EBL model due to the meaningless strengthening effect of these partially peeled CFRP sheets. Moreover, for the sake of safety, data of the two specimens (C30-20-5 and C50-40-10) with insufficient development of bonding properties were also excluded from the modeling process. EBL model considering corrosion was obtained by linear fitting in this study, as shown in Equation (5).

(5) $$L_{e}=\sqrt{E_{f}\cdot t_{f}/\sqrt{f_{c}^{’}}}+6.31\cdot \eta ,\;(\eta \leq 12\%)$$

Correlation coefficient (R^2^) between Equation (5) and experimental data was 0.94. This model can be used to calculate the EBL of CFRP sheets strengthening RC elements with corrosion degree of less than 12%, thus providing a reference value for deciding the actual length of the CFRP sheet.

### 4.2. Bond Stress-Slip Relationships Considering Corrosion

#### 4.2.1. Combined Effect on Bond Parameters

Dai et al. [32] proposed an e-function (Equation (6)) model to predict strain and slip relationships, the reliability of which has also been confirmed by other scholars [33,34].

(6) ε=f(s)=A(1−exp(−Bs))

(7) $$P_{u}=E_{f}A_{area}\varepsilon_{\max}=E_{f}A_{area}\underset{s\to \infty }{\lim}A(1-\exp(-Bs))=E_{f}A_{area}A$$

where A means the maximum strain ($\varepsilon_{\max}$) with sufficient bond length, as indicated in Equation (7). B is defined as the brittleness index, which controls the shape of the bond-slip curve [35]. $A_{\mathrm{area}}$ is the cross-sectional area of CFRP sheets.

**Figure 8:**
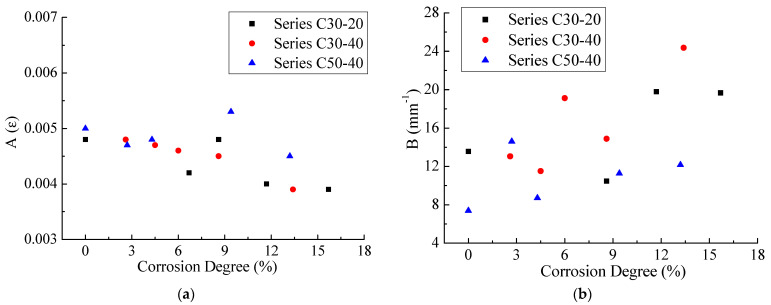
Effects of concrete compressive strength, concrete cover thickness and corrosion degree on (**a**) coefficient A and (**b**) coefficient B.

Values of bond parameters A and B with correlative factors R^2^ are shown in Table 3. The dependency of these two parameters on corrosion degree and concrete strength, as well as concrete cover thickness, is shown in Figure 8. It should be noted that due to the small value of R^2^, specimens C30-20-5 and C30-40-7 were excluded in the modeling process. A decreases with the increase in corrosion degree, while the reverse is true for B. Concrete cover thickness has little effect on A and B. Specimens with higher concrete strength have higher value of A and lower value of B, and this result is consistent with the conclusion proposed by López-González et al. [36]. Therefore, coefficients A and B are greatly affected by corrosion degree and concrete strength and are less affected by the thickness of the concrete cover.

#### 4.2.2. Improvement of Bond-Slip Model Considering Corrosion

Dai et al. [32] further derived a bond stress-slip model that contains two coefficients  and $G_{f}$ (Equation (8)), where $G_{f}$ is expressed by A (Equation (9)). They have studied the impact of three items (concrete strength, fiber forced plastic stiffness and adhesive) on $G_{f}$ and two items (fiber forced plastic stiffness and adhesive) on B, and found the little dependence of B and $G_{f}$ on CFRP stiffness. However, they did not consider the combined impact of concrete strength and corrosion degree on B and $G_{f}$ which has been proved in Section 4.2.1.

(8) $$\tau =E_{f}t_{f}\cdot d\varepsilon /dx=E_{f}t_{f}\cdot df(s)/ds\cdot d\varepsilon /dx=E_{f}t_{f}\cdot df(s)/ds\cdot \varepsilon \;=A^{2}BE_{f}t_{f}\exp(-Bs)(1-\exp(-Bs))\;=2BG_{f}\exp(-Bs)(1-\exp(-Bs))$$

(9) $$G_{f}=\int_{0}^{\infty }\tau ds=0.5A^{2}E_{f}t_{f}$$

**Figure 9:**
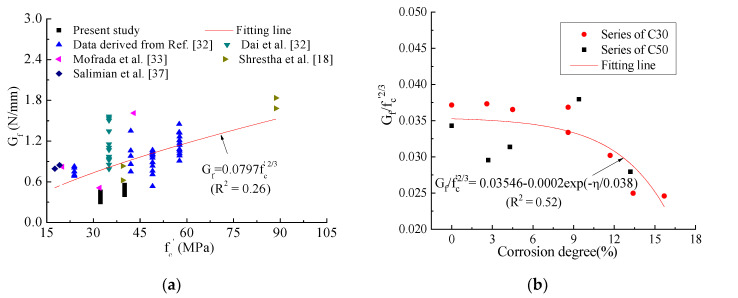

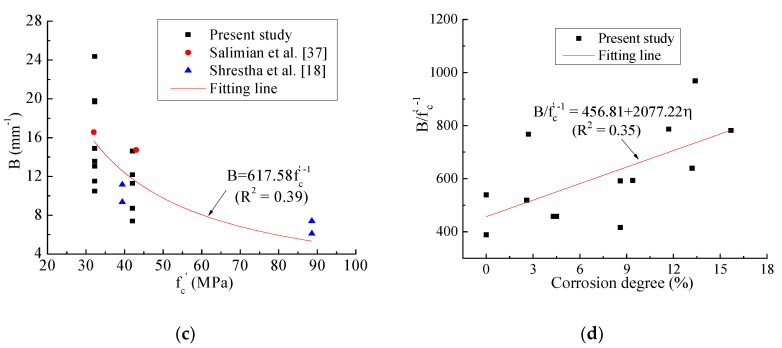
Combined effect of concrete strength and corrosion on $G_{f}$ and B. (**a**) (modified from Dai et al. [23]) Effect of concrete strength on $G_{f}$ [18,32,33,37]; (**b**) Combined effect of concrete strength and corrosion degree on $G_{f}$; (**c**) Effect of concrete strength on B [18,37]; (**d**) Combined effect of concrete strength and corrosion degree on B.

In this study, in the case of using common adhesive, i.e., without considering the factor of adhesive, the expressions of B and $G_{f}$ are proposed with combined influence of concrete grade and corrosion degree. Dependency of B and $G_{f}$ on concrete strength was established based on more experimental databases, as shown in Figure 9a,c. It should be pointed out that B and $G_{f}$ are not directly given in these collected references, so the bond stress-slip curves were used to fit with Equation (8) to obtain B and $G_{f}$. Due to the limited experimental research on bond properties between CFRP sheets and corrosion-cracked concrete, the effect of corrosion on B and $G_{f}$ was analyzed only based on the experimental data in this study, as shown in Figure 9b,d. Expression for B and $G_{f}$ was obtained by nonlinear fitting, as shown in Equations (10) and (11).

(10) $$G_{f}=f_{c}^{’2/3}(0.03456-0.0002exp(-\eta /0.038))$$

(11) $$B=f_{c}^{’-1}(456.81+2077.22\cdot \eta )$$

With Equations (8), (10) and (11), the bond stress-slip relationship of CFRP sheets and corrosion-induced cracking concrete interface can be obtained (see Equation (12)).

(12) $$\begin{array}{l} \tau =2f_{c}^{’-1/3}(0.03456-0.0002\exp(-\eta /0.038))(456.81+2077.22\cdot \eta )\\ \cdot \exp(-(f_{c}^{’-1}(456.81+2077.22\cdot \eta ))\cdot s))(1-\exp(-f_{c}^{’-1}(456.81+2077.22\cdot \eta ))\cdot s)) \end{array}$$

#### 4.2.3. Verification of Proposed Bond Stress-Slip Model with Experimental Results

**Figure 10:**
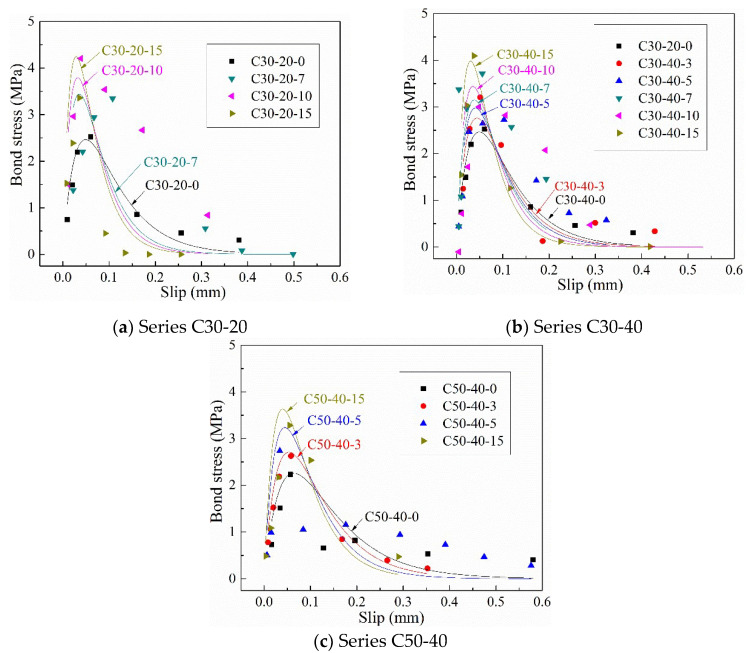
Comparison between the proposed bond stress-slip model and experimental results.

Local slip at section i ($S_{i}$) between CFRP sheets and concrete can be calculated by Equation (13)

(13) $$S_{i}=\Delta x/2\cdot (\varepsilon_{0}+2\sum_{j=1}^{i-1}\varepsilon_{j}+\varepsilon_{i})$$

where $\varepsilon_{0}$ and $\varepsilon_{j(j=1,i)}$ are the strain of free end and the j th gauge, respectively. Figure 10 shows the bond shear stress-slip at 15 mm from the loaded end in this test. The proposed bond stress-slip law (Equation (12)) is also drawn with solid lines with different colors to distinguish different corrosion degrees. As shown in Figure 10, the proposed model has high prediction accuracy, especially the peak point, i.e., the maximum bond stress and the corresponding slip. Except for the specimens with a low corrosion degree (3%) or no corrosion, the descending portion of the curve is more conservative than the experimental value. It can be explained that the accumulated corrosion medium on the CFRP-concrete interface affects the deformation of CFRP sheets, resulting in large strain values at local measurement points. Besides, the maximum bond stress increases with the increase in corrosion degree, which is similar to the result proposed by Pan et al. [38]. Although concrete strength has little effect on the maximum bond strength, as mentioned earlier, it has an obvious positive effect on the slip corresponding to the maximum bond stress.

**Figure 11:**
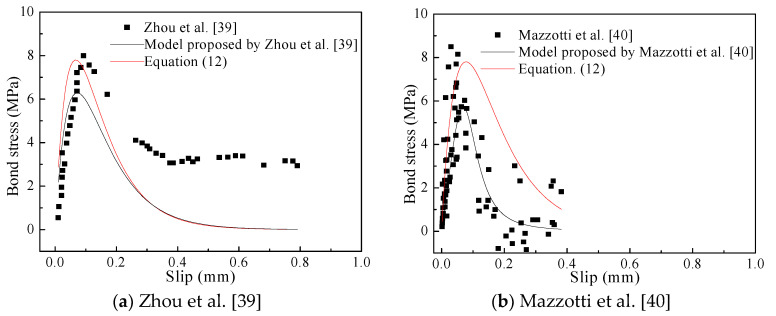
Verification of the proposed model: (**a**) Zhou et.al [39]; (**b**) Mazzotti et.al [40].

The accuracy and applicability of the proposed model were further verified by other test data which were not used for the modeling process (Figure 11). These experimental data are the bond shear stress-slip relationship at the loaded end of non-corrosive specimens, and the interface laws proposed by these researchers based on their own experimental data are also plotted for comparison with the developed model in this study. As shown in Figure 11, the proposed model could more accurately predict the bond stress-slip relationship than the existing interface laws, especially for the peak point. But the prediction precision of the proposed model may be further verified and improved by more additional data from further research on the bond behavior between CFRP sheets and corrosion-damaged concrete. 

## 5. Conclusions

Combined effects of corrosion degree and concrete strength, as well as concrete cover thickness on bond behavior of CFRP to the corrosion-cracked concrete interface were investigated by an accelerated corrosion test and a double shear lap test with a developed self-balancing device in this study.

There are three stripping modes of CFRP sheets with respect to different corrosion degrees. Non-corrosive specimens and those with corrosion degrees lower than 5% showed failure mode A in which CFRP sheets were completely stripped. Three specimens with intermediate corrosion degree (6.2%, 6.7% and 9.4%) displayed failure mode B, i.e., large-area peeling of CFRP sheets, which leads to the unbalance of self-balancing device. Severely corroded specimens showed mode C in which CFRP sheets were torn along the concrete crack and peeled off in pieces.

When corrosion degree is less than 12%, EBL has a positive linear correlation with corrosion degree, but barely depends on concrete grade and concrete cover thickness. When corrosion degree is higher than 12%, the characteristic of failure mode C and the adverse effects of immersion condition, as well as the accumulation of corrosion medium make the EBL lower than that of the benchmark specimens.

An in-depth data analysis shows that corrosion degree and concrete strength obviously affect two bond parameters of the CFRP-concrete interface, while the impact of concrete cover thickness can be ignored. A nonlinear degenerate interface law was proposed and verified with the test data in this study and also the collected data. Specifically, the maximum bond stress increases with the increase in corrosion degree. Besides, concrete strength has little effect on the maximum bond stress, whereas it has an effect on the slip corresponding to this stress.

## Figures and Tables

**Table 1:** Specimen labels.

Series		C30-20	C30-40	C50-40
Concrete strength degree		C30	C30	C50
Concrete cover (mm)		20	40	40
Target corrosion degree (%)	0	C30-20-0	—	C50-40-0
	3	C30-20-3	C30-40-3	C50-40-3
	5	C30-20-5	C30-40-5	C50-40-5
	7	C30-20-7	C30-40-7	C50-40-7
	10	C30-20-10	C30-40-10	C50-40-10
	15	C30-20-15	C30-40-15	C50-40-15

**Table 2:** Mechanical properties of CFRP and adhesive.

	Type	Thickness (mm)	Elastic Modulus (GPa)	Tensile Strength (MPa)
CFRP	TZ-300g	0.167	239	3723
Adhesive	DA-T	0.5–0.9	3.01	44.6

**Table 3:** Test results of double shear lap test.

ID	η (%)	$P_{cr}\;(\mathrm{kN})$	$P_{u}\;(\mathrm{kN})$	EBL (mm)	A	B	$G_{f}$	${R^{2}}^{\;}$	Failure Mode
C30-20-0	0	24	30.4	87.0	0.0048	13.56	0.4541	0.97	Mode A
C30-20-5	6.7	24	27.3	97.8	0.0042	28.37	0.3487	0.56	Mode B
C30-20-7	8.6	25	30.0	132.0	0.0048	10.47	0.4503	0.98	Mode A
C30-20-10	11.7	20	27.1	160.8	0.0040	19.79	0.3690	0.98	Mode A(CFRP fiber teared)
C30-20-15	15.7	20	26.0	58.0	0.0039	19.66	0.3004	0.94	Mode C
C30-40-3	2.6	20	30.2	113.6	0.0048	13.05	0.4560	0.98	Mode A
C30-40-5	4.5	20	28.8	121.3	0.0047	11.51	0.4465	0.99	Mode A
C30-40-7	6.0	15	27.9	128.0	0.0046	19.11	0.4132	0.69	Mode B
C30-40-10	8.6	20	28.5	136.5	0.0045	14.88	0.4077	0.99	Mode C
C30-40-15	13.4	20	27.2	103.6	0.0039	24.36	0.3051	0.97	Mode C
C50-40-0	0	20	31.3	77.9	0.0050	7.39	0.5009	0.98	Mode A
C50-40-3	2.7	24	29.6	97.5	0.0047	14.60	0.4315	0.98	Mode A
C50-40-5	4.3	15	32.1	115.9	0.0048	8.71	0.4579	0.94	Mode A
C50-40-10	9.4	15	26.2	105.0	0.0053	11.28	0.5543	0.99	Mode B(CFRP fiber teared)
C50-40-15	13.2	15	27.5	53.8	0.0045	12.16	0.4077	0.99	Mode C

## Data Availability

The data presented in this study are available upon request from the corresponding author.
